# Consumption of dairy, fruits and dark green leafy vegetables is associated with lower risk of adverse pregnancy outcomes (APO): a prospective cohort study in rural Ethiopia

**DOI:** 10.1038/s41387-018-0060-y

**Published:** 2018-09-20

**Authors:** Taddese A. Zerfu, Elisabete Pinto, Kaleab Baye

**Affiliations:** 1 College of Medicine and Health Sciences and Referral Hospital, Dilla University, Dilla, Ethiopia; 2 Maternal and Child Well Being Unit, African Population and Health Research Center, Nairobi, Kenya; 3 Center for Food Science and Nutrition, College of Natural and Computational Sciences, Addis Ababa University, Addis Ababa, PO Box 1176 Ethiopia; 4 CBQF - Centro de Biotecnologia e Química Fina - Laboratório Associado, Escola Superior de Biotecnologia, Universidade Católica Portuguesa, Rua Arquiteto Lobão Vital, 172, Porto, 4200-374 Portugal; 5 EPIUnit - Instituto de Saúde Pública, Universidade do Porto, Rua das Taipas, n° 135, Porto, 4050-600 Portugal

## Abstract

**Background:**

Poor maternal nutrition during pregnancy is a leading modifiable risk factor associated with risks of adverse pregnancy outcomes (APO). Nevertheless, there is paucity of evidence if consumption of some food groups is associated with lower risk of APO, particularly in low-income settings. We aimed to determine whether consumption of some food groups is associated with lower risk of APOs such as: preterm birth (PTB), low-birth weight (LBW), and stillbirth in rural Central Ethiopia.

**Methods:**

A multi-center (8 health centers) prospective cohort study, enrolling 432 pregnant women during their initial antenatal care visit, was employed. All mothers were then followed monthly (for a total of four visits) from enrollment to delivery. Midwives in respective health centers assessed dietary diversity using the Women’s individual dietary diversity score and evaluated birth outcomes following standard procedures. Logistic regression models were run to predict association of food groups with the APO.

**Findings:**

Out of the 374 pregnant women who completed the study, one in five [74 (19.8%)] experienced at least one of the APO: 34 (9.1%) gave birth to LBW babies, 51(13.6%) had PTB and 17 (4.5%) experienced stillbirth. Poor or inconsistent consumption (<¾ assessments) of dark green leafy vegetables (adjusted odds ratio (AOR) = 2.01; 95% confidence interval (CI): 1.04–3.87), dairy products (AOR = 2.64; 95% CI: 1.11–6.30), and fruits and vegetables (AOR = 2.92; 95% CI: 1.49–5.67) were independently associated with higher APO risks. Whereas, being nonanemic at term (AOR = 0.24; 95% CI: 0.12–0.48) was independently associated with lower APO risks.

**Conclusions:**

Poor or inconsistent consumption of dairy, dark green leafy vegetables and fruits were associated with higher risk of APOs. While community-based trials and mechanistic studies are needed to substantiate these findings, efforts to promote dietary diversity through increased consumption of fruits, vegetables and dairy may be beneficial in this and similar settings.

## Introduction

In the last couple of decades, a substantial progress in child mortality was made worldwide^1,2^. Some of the world’s poorest countries have managed to cut child mortality rates by >50% in just a couple of decade’s time^2^. The mortality rate of under-five children has also declined substantially from 12.6 (12.4–12.8) in 1990 to 5.6 (5.4–6.0) million deaths in 2016^1,2^. Despite these achievements, 5.6 million under five children continue to die every year mainly from preventable causes. Majority of child deaths occur during the neonatal period (first one month). About one million of neonates die on the first day and another close to one million die in the first week^3^.

Adverse outcomes of pregnancy, mainly: preterm birth (PTB), low-birth weight (LBW), and stillbirth continue to be major causes of neonatal and under five child deaths, worldwide^4,5^. In 2015 alone, close to 15 million premature (<37 completed weeks of gestation) babies were born; of which, more than a million died during the neonatal period due to complications, and those surviving often experienced lifetime of impairments^5,6^.

In Ethiopia, recent estimates suggest that prenatal death happens in 46 out of 1000 pregnancies and about 11% of newborns have LBW (7 and 8). These estimates rank Ethiopia among the ten top countries with the highest global burden of maternal (fifth) and neonatal (sixth) deaths, as well as stillbirth (seventh) incidence rates.

Poor nutrition of women during pregancy is among the leading modifiable risk factors associated with elevated APO risks^10–15^. Evidence is accumulating that maternal dietary patterns during pregnancy is associated with some APO risks. Studies from Europe^16–19^, New Zealand^20^, and Brazil^21^ showed that maternal adherence to certain dietary patterns reffered as “western”, “prudent”, or “traditional” diets were positively associated with birth weight, offspring bone size, bone mineral density, and forearm fractures; with additional protective effect against risks of having infants of small for gestational age. However, the associations between dietary diversity on APO risks remains unclear as some studies show heightened or null-effects^22,23^. Besides, many of the studies investigating such associations are mainly from high-income settings and hence studies from resource-poor settings are urgently needed. Therefore, in this study, we aimed to determine the association between consumption of some food groups with APO risks.

## Methods

This is part of a larger study that aimed to investigate dietary patterns during pregnancy and several outcomes. Detailed information about the larger study and the methods applied are reported elsewhere^24^. Briefly, the study employed a prospective cohort follow-up study design enrolling 374 pregnant women from their initial antenatal care (ANC). All mothers were then followed monthly (for a total of four visits) from enrollment to delivery.

The study was conducted in eight health centers randomly selected from four rural districts of Oromia Regional State, Ethiopia. The four rural districts were selected purposively to represent all agro climatic areas and socio-cultural aspects of the zone. Pregnant women were enrolled to the study according to their exposure to diversify (exposed) and nondiversified (unexposed) diets.

We used the following criteria to include mothers to the study: permanent residence (weather she lived at least for 6 months in the area or not), apparently healthy (having no known medical, surgical, or obstetric problems), and mother willing to attend the monthly ANC visits.

We collected socio-economic data including: age, educational status, land ownership and others using questionnaire-based and face-to-face interviews. Midwives in each health center collected anthropometric data during enrollment and follow-up ANC visit. Weight was measured to the nearest 100 g using electronic scales (Salter Brecknell) with a weighing capacity of 10–140 kg following the standardized procedures recommended by World Medical Association^25^ and World Health Organization^26^. Portable devices equipped with calibrated and standardized height gauges (SECA 206 Body meter) were used to measure the height of mothers to the nearest mm during each visit. Using a nonstretch measuring, midwives also measured the Mid-upper arm circumference (MUAC).

Women dietary diversity scores (WDDS) was collected monthly using a four 24 h dietary recall from enrollment to delivery. According to FAO, the WDDS has nine food groups that include: (i) cereals, (ii) roots and tubers; (iii) vitamin A rich fruits and vegetables; (iv) other fruit and vegetables; (v) legumes and nuts; (vi) meat, poultry, and fish; (vii) fats and oils; (viii) dairy; and (ix) eggs.

**Fig. 1:**
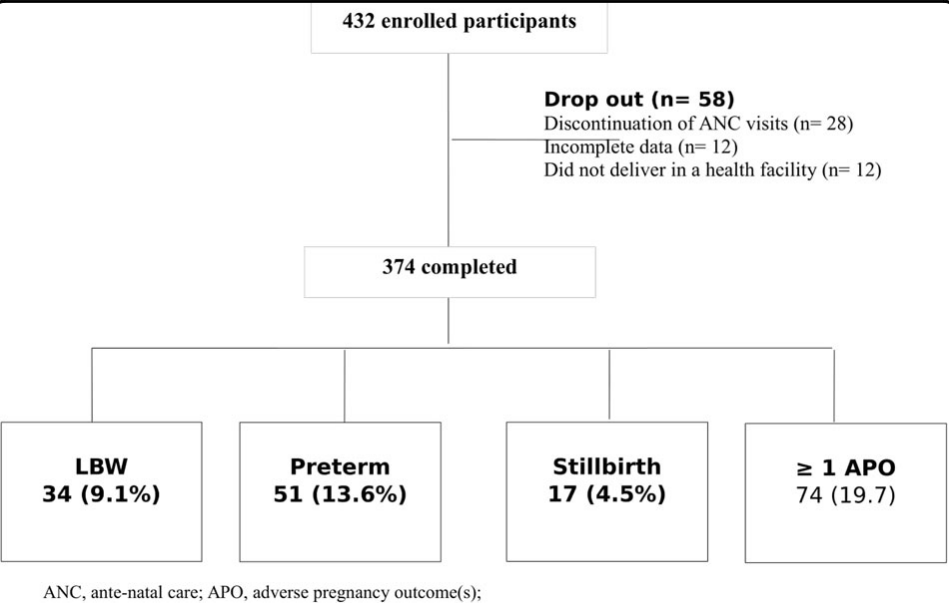
Study participant flowchart— prospective cohort of pregnant mothers, in rural Arsi, Central Ethiopia

A woman was considered to be either in the diversified (“consumers”) or a nondiversified (“nonconsumers”) group, based on data computed from the WDDS. Accordingly, if a woman completed the four monthly visits and remained in same group for at least three of the four visits, she was considered to be part of the group. Women who missed a visit or changed dietary diversity group more than once, were excluded from the analysis as shown in Fig. 1.

### Outcome assessment

The midwives were trained on how to take hemoglobin levels using a portable HemoCue (AB Leo Diagnostics, Helsinborg, Sweden). Accordingly, hemoglobin levels were taken twice at enrollment and at term for every women. The readings were adjusted for altitude^20^, and women whose altitude adjusted hemoglobin values below 11.0 g/dl were considered to be anemic^26^. They also recorded birth weight to the nearest 100 g, and assessed stillbirth (a baby born with no signs of life at birth after 24 completed weeks of gestation) as well as PTB (delivery < 37 weeks of gestation), immediately after delivery. The midwives have also estimated gestational age of pregnancy, by combing data from the women’s report of last menstrual period and performing fundal palpation^27^.

### Statistical analyses

Data entry and cleaning was conducted using Epi-data and SPSS (version 20.0) statistical software (3.1). Kolmogorov–Smirnov test was used to test normality of continuous variables. A log-binomial model was run to adjust baseline group differences of key variables including MUAC, educational level, hemoglobin, age, height, and gestational age.

We reported Adjusted ORs with 95% CI values for regression models run. Differences with *p* values < 0.05 were considered statistically significant. A bivariate and multivariate logistic regression analyses were to test the association of food groups consumed and APOs.

Furthermore, false discovery rate adjustment was performed using the Benjamini–Hochberg (B–H) procedure to reduce the inflation of type 1 error due to multiple comparisons. The following formula was used to calculate B–H critical values for each *p* value:

Benjamini–Hochberg (B–H) = (*i*/*m*)*Q*; where,

*i* = the individual *p* value’s rank,   *m* = total number of tests, and*   Q* = the false discovery rate (set at 15%).

We then compared our original *p* values to the critical B–H values, and all *p* values were < 0.05, with the highest value obtained for dark green leafy vegetables (*p* = 0.043).

## Results

We enrolled a total of 432 (216 from each group) eligible pregnant women who fulfilled the inclusion criteria. Amongst of these, 374 completed the study. This yielded an overall dropout rate of 13.4%, which was balanced across groups. The main reasons for dropout were: discontinuation of ANC visits (*n* = 28), shift from study group (*n* = 12), and delivery at home or away from index health facility (*n* = 18) (Fig. 1).

Close to 1 in every 5 (19.8%) women, of those who remained in the study, experienced at least 1 of the APOs. As such, 51 (13.6%) gave birth to premature baby, 34 (9.1%) had a LBW baby, and 17 (4.5%) had a stillbirth babies who died at, or immediately after birth, Fig. 1.

Table 1 presents selected socio-demographic, nutritional and anthropometric characteristics of pregnant women by their APO experience. Likewise, only close to half (*n* = 159; 42.5%) of the women completed primary education, and majority (*n* = 147; 39.3%) were in the early 20s^20–24^ by age category, and 241 (65.1%) had ≥4 ANC visits. A sizable proportion 166(44.9%) of women owned two or more hectares of land. Hematologic investigations showed that more than two-third (71.4 % at enrollment and 67.9 % at term) were nonanemic.

**Table 1:** Selected socio-demographic, anthropometric, and nutritional characteristics of pregnant women in rural Ethiopia, stratified by adverse pregnancy outcomes (*n* = 374)

Selected characteristics	All (*n* = 374)	LBW (*n* = 34)	Preterm (*n* = 51)	Stillbirth (*n* = 17)	APO (*n* = 74)
*Maternal age (y)*					
<20	34 (9.1)	1 (2.9)	1 (2)	0	2(2.7)
20–24	147 (39.3)	12 (35.5)	13 (25.5)	2 (11.8)	23 (31.1)
25–29	130 (34.8)	15 (44.1)	27 (52.9)	8 (47.1)	33 (44.6)
≥30	63 (16.8)	6 (17.6)	10 (19.6)	7 (41.2)	16 (21.6)
*Ethnicity*					
Oromo	301 (80.5)	27 (79.4)	33 (64.7)	13 (76.5)	53 (71.6)
Amhara	52 (13.9)	5 (14.7)	10 (19.6)	2 (11.8)	11 (14.9)
Guraghe	17 (4.5)	2 (5.9)	8 (15.7)	29 (11.8)	10 (13.5)
Other	4 (1.1)	0 (0)	0 (0)	0 (0)	0 (0)
*Educational status*					
Unable to read and write	120 (32.1)	7 (20.6)	18 (35.3)	8 (47.1)	25 (33.8)
Read and write only	95 (25.4)	17 (50)	16 (31.4)	5 (29.1)	26 (35.1)
Primary education	31 (8.3)	2 (5.9)	7 (13.7)	2 (11.8)	7 (9.5)
Secondary or above	128 (34.2)	8 (23.5)	6 (11.7)	2 (11.8)	16 (21.6)
*Land size (hectare)*					
<One	166 (44.9)	8 (23.5)	27 (52.9)	9 (52.9)	30 (40.5)
One–two	136 (36.4)	19 (55.9)	18 (35.3)	4 (23.5)	33 (44.6)
>Two	70 (18.7)	7 (20.6)	6 (11.8)	4 (23.5)	11(14.9)
*Hemoglobin (Baseline)*					
Anemic (Hb < 110 g/l)	107 (28.6)	22 (64.7)	28 (54.9)	8 (47.1)	39 (52.6)
Nonanemic	267 (71.4)	12 (64.3)	23 (45.1)	9 (52.9)	35 (47.3)
*Hemoglobin (term)*					
Anemic (Hb < 110 g/l)	121 (32.1)	27 (79.4)	33 (64.7)	11 (64.7)	48 (64.8)
Nonanemic	253 (67.9)	7 (20.6)	18 (35.3)	6 (35.3)	26 (55.2)
*MUAC (cm)*					
Malnourished (<23)	173 (46.3)	26 (76.5)	35 (68.6)	11 (64.7)	49 (66.2)
Well-nourished (≥23)	201 (53.9)	8 (23.5)	16 (31.4)	6 (35.3)	25 (33.8)

*MUAC* mid-upper arm circumference

Bivariate analyses results showed that pregnant women who had poor consumption of dark green leafy vegetables, vitamin A-rich foods, other fruits and vegetables, milk and milk products, experienced a higher risk of APO, compared to those women who reported better consumption. Nevertheless, dark green leafy vegetables and dairy products remained to be independent predictors of APO risks in the final multivariate logistic regression analysis, Table 2. As such, pregnant women with poor consumption of dark green leafy vegetables had a 92% (AOR = 1.92; 95% CI: 1.04–3.58) added risk of experiencing at least one of the APO relative to consumers. Similarly, those pregnant women who had poor consumption of dairy products were >3× more (AOR = 3.35; 95% CI: 1.48–7.60) at risk of APO compared to those who consumed dairy products more regularly (Table 2).

**Table 2:** Association between consumption of specific food groups with adverse pregnancy outcomes in Central Arsi, rural Ethiopia (*n* = 374)^a^

Food groups consumed	Number (%)	Adverse pregnancy outcome (APO)			
		COR (95% CI)	AOR (95% CI)	*p* Values	(*i*/*m*)*Q*^b^
*All types of fruits and vegetables*					
Yes	212 (56.7)	1	1	0.010	0.015
No	162 (43.3)	3.24 (1.89, 5.54)^b^	2.92 (1.49, 5.67)^b^		
*Milk and products*					
Yes	113 (30.2)	1	1	0.023	0.030
No	261 (69.8)	3.35 (1.65, 6.79)^b^	2.64 (1.11, 6.30)^b^		
*Dark green leafy vegetables*					
Yes	146 (39)	1	1	0.043	0.045
No	228 (61)	2.31 (1.29, 4.31)^b^	2.01 (1.04, 3.87)^b^		
*Starchy staples*					
Yes	374 (100)	–	–		
No	0 (0)	–	–		
*Meat and fish*					
Yes	17 (4.5)	1	1	0.054	0.060
No	357 (95.5)	1.89 (0.42, 8.47)	0.50 (0.07, 3.84)		
*Organ meat*					
Yes	23 (6.1)	1	1	0.067	0.075
No	351 (93.9)	2.71 (0.62, 11.82)	1.21 (0.20, 7.27)		
*Vitamin A rich foods*					
Yes	103 (27.5)	1	1	0.112	0.090
No	271 (72.5)	2.01 (1.05, 3.84)^b^	1.23 (0.57, 2.65)		
*Other fruits and vegetables*					
Yes	83 (22.2)	1	1	0.230	0.105
No	291 (77.8)	2.75 (1.26, 5.99)^b^	1.48 (0.57, 3.84)		
*Legumes*					
Yes	333 (89)	1	1	0.340	0.120
No	41 (11)	1.16 (0.53, 2.55)	1.39 (0.56, 3.49)		
*Eggs*					
Yes	71 (19)	1	1	0.551	0.135
No	303 (81)	1.88 (0.89, 3.99)	0.59 (0.21, 1.66)		
*Animal source foods (ASF)* ^c^					
Yes	141 (37.7)	1	1	0.643	0.150
No	233 (62.3)	2.85 (1.54, 5.25)^b^	1.46 (0.69, 3.06)		

^a^Logistic regression adjusted for baseline MUAC and hemoglobin concentrations; APO, adverse pregnancy outcomes

^b^Benjamini–Hochberg critical value *P < 0.05

^c^All ASF (meat, dairy, eggs, and fish) are included

Among maternal nutritional characteristics run the multivariate logistic regression analysis, only anemia at term was found to be independently associated with higher APO risks. Accordingly, nonanemic women whose altitude adjusted hemoglobin level was higher than 11 mg/dl at term had a 77% reduced risk (AOR = 0.23; 95% CI: 0.11–0.45) of experiencing APO compared to those who were anemic (Table 3).

**Table 3:** Association between anthropometric measures and hemoglobin concentration with adverse pregnancy outcomes in central Arsi, rural Ethiopia

Nutritional Characteristics	Number (%)	Adverse Pregnancy Outcome (APO)	
		COR(95% CI)	AOR (95% CI)
Weight gained (kg)			
<6	33 (8.8)	1	1
6–9	155 (41.4)	0.98 (0.41, 2.37)	1.23 (0.46, 3.32)
9.1–12.5	147 (39.3)	1.64 (0.66, 4.07)	1.78 (0.64, 4.99)
>12.5	39 (10.4)	2.80 (0.76, 10.37)	2.60 (0.63, 10.78)
MUAC (cm)			
<21	90 (24.1)	1	1
21–23	171 (45.7)	0.31 (0.15, 0.64)	1.39 (0.72, 2.70)
23+	113 (30.2)	0.61 (0.31, 1.21)	1.72 (0.76, 3.86)
Hemoglobin (g/dl) - at term			
<11	121 (32.4)	1	1
≥11	253 (67.6)	0.17 (0.10, 0.30)	**4.56 (2.25, 9.25)**^a^*

^a^logistic regression adjusting for baseline hemoglobin concentrations; *P < 0.05

## Discussion

We employed a prospective cohort design to examine the associations between dietary patterns during pregnancy with APO risks in a resource-limited setting of rural central Ethiopia. Anemia at term and poor consumption of dairy, fruits, and vegetables (dark green leafy vegetables) were associated with higher risk of experiencing at least one of the APOs: LBW, preterm, and stillbirth.

Although 60% of preterm births^6^, 98% of stillbirths^5^ and 96.5% of LBW infants^28^ occur in low-income settings, mainly in Africa and South Asia, there is a critical shortage of evidence investigating the association of APO with dietary patterns during pregnancy. Most of the existing studies in Ethiopia are cross-sectional^7–9^; and hence, ill-fitted to relate outcomes with dietary patterns. However, another study in Ethiopia showed that dietary diversity ≥4 (out of 9 food groups) has been correlated with lower risk of maternal anemia, preterm birth, and low birth weight babies^24^, but which food groups are associated with reduced risk remained unknown.

In the present analysis, our multivariate analysis suggests that poor intake of dairy and dark green leafy vegetables during pregnancy were found to be independent predictors of higher APO risks. Pregnant women who had poor consumption of dairy, fruits and vegetables had nearly two to four fold increased risks of APO, compared to those who consumed either of these food items. This is in line with the Iranian^29^, Spanish^30^, and Indian^31^ prospective studies that enrolled and followed pregnant women all showing reduced risk of APOs with increasing consumption of dairy, green leafy vegetables, and fruits.

Many factors can explain the role of dairy consumption in reducing the risk of APO. First, dairy is a good source of essential nutrients, including calcium. The role of calcium in preventing preeclampsia, a leading contributor to APO, is well-documented^32^. Second, proteins and growth hormones provided by dairy can support fetal growth and thus may increase birth weight. Beyond its nutritional contribution, milk can also promote anabolism and serve as an endocrine signaling system for postnatal growth by activating the nutrient-sensitive kinase mTORC1^33,34^, thus increasing gestational age, placental, and fetal weight. On the other hand, fruits and vegetables have been found to have a protective role against APO. Perhaps, this role can be conferred by micronutrients and antioxidants found in these foods, which contribute to optimal immune and placental functions, and besides contribute to fetal growth^35,36^

The study had limitations that require curiosity and consideration when interpreting the findings. Firstly, unlike preconception nutrition is associated with pregnancy outcomes, we were only able to follow the women starting from their second trimester, mainly related to maternal habit of late ANC onset in the country^7^. Secondly, in spite of our efforts to adjust for baseline differences computing APOs risks, the effect of hidden factors in the model may not be completely controlled. Our study was not powered enough to evaluate associations of consumption of each food groups with individual APOs. Besides, given that this is a health-facility based study, our sampling might be subject to favoring women with better access to health facilities.

Notwithstanding the above limitations, our study highlights that consistent consumption of dairy products, fruits, and vegetables (dark green leafy vegetables) were found to be independently associated with APO risks. While community-based trials and mechanistic studies are needed to establish causal relationships, promotions of the consumption of these food groups in Ethiopia and other similar settings may be beneficial.
